# Acetotrophic Activity Facilitates Methanogenesis from LCFA at Low Temperatures: Screening from Mesophilic Inocula

**DOI:** 10.1155/2019/1751783

**Published:** 2019-05-02

**Authors:** Suniti Singh, Johanna M. Rinta-Kanto, Riitta Kettunen, Piet Lens, Gavin Collins, Marika Kokko, Jukka Rintala

**Affiliations:** ^1^Faculty of Engineering and Natural Sciences, Tampere University, Tampere, Finland; ^2^IHE, Institute for Water Education, Westvest 7, 2611AX Delft, Netherlands; ^3^National University of Ireland Galway, University Road, Galway H91 TK33, Ireland

## Abstract

The inoculum source plays a crucial role in the anaerobic treatment of wastewaters. Lipids are present in various wastewaters and have a high methanogenic potential, but their hydrolysis results in the production of long chain fatty acids (LCFAs) that are inhibitory to anaerobic microorganisms. Screening of inoculum for the anaerobic treatment of LCFA-containing wastewaters has been performed at mesophilic and thermophilic conditions. However, an evaluation of inocula for producing methane from LCFA-containing wastewater has not yet been conducted at low temperatures and needs to be undertaken. In this study, three inocula (one granular sludge and two municipal digester sludges) were assessed for methane production from LCFA-containing synthetic dairy wastewater (SDW) at low temperatures (10 and 20°C). A methane yield (based on mL-CH_4_/g-COD_added_) of 86-65% with acetate and 45-20% with SDW was achieved within 10 days using unacclimated granular sludge, whereas the municipal digester sludges produced methane only at 20°C but not at 10°C even after 200 days of incubation. The acetotrophic activity in the inoculum was found to be crucial for methane production from LCFA at low temperatures, highlighting the role of *Methanosaeta* (acetoclastic archaea) at low temperatures. The presence of bacterial taxa from the family *Syntrophaceae* (*Syntrophus* and uncultured taxa) in the inoculum was found to be important for methane production from SDW at 10°C. This study suggests the evaluation of acetotrophic activity and the initial microbial community characteristics by high-throughput amplicon sequencing for selecting the inoculum for producing methane at low temperatures (up to 10°C) from lipid-containing wastewaters.

## 1. Introduction

High rate anaerobic treatment is an efficient solution to treat wastewaters without expending energy for aeration and to simultaneously produce a bioenergy source as methane. Mesophilic conditions have been commonly applied for the anaerobic treatment of wastewaters due to the optimal growth temperature range of the microbial consortia [1]. However, numerous wastewaters, including domestic and industrial, are discharged at temperatures of 20°C or lower, and their anaerobic treatment at these low temperatures could improve the net energy gain from the treatment process.

Over 700 billion metric tons of milk is produced annually worldwide, which leads to the generation of huge volumes of dairy wastewaters [2, 3]. These dairy effluents constitute high amounts of lipids (35-500 mg/L) along with carbohydrates and proteins [4], and the presence of each of these constituents poses specific challenges. Lipids have a higher methanogenic potential than carbohydrates or proteins [5], but their anaerobic treatment is challenging due to the synergistic inhibitory effects of their hydrolysis by-products [6–10]. Single compounds [11, 12] as well as industrial wastewaters [13–16] have been treated anaerobically at low temperatures. Yet, the anaerobic treatment of lipids at low temperatures (≤20°C) remains understudied.

During lipolysis, the lipids hydrolyze to long chain fatty acids (LCFAs) which are inhibitory to acidogens, acetogens, and methanogens, even at low concentrations [6–9, 17]. For example, at 21°C, 30 mg/L of linoleic acid, 30 mg/L of oleic acid, and 10 mg/L of stearic acid inhibit methane production from 100 mg/L of acetate [18, 19]. Additionally, LCFA impedes methane production by imposing mass-transfer limitations and preventing aggregation of acetogens and methanogens [20, 21]. Although the individual and synergistic inhibitory effects of LCFA (oleic, stearic, and linoleic acids) on hydrogen, glucose, and butyrate fermentation have been studied in batch at 21°C, the methane production was not reported [7] which obscures the potential use of LCFA as a methanogenic precursor under psychrophilic conditions. Investigations on the anaerobic treatment of dairy wastewaters (4 g-COD/L) with a low fat content of 40 mg-COD/L (typically <1% of the total chemical oxygen demand) have been conducted previously at temperatures as low as 10 and 15°C [22]. Very recently, the knowledge on lipid treatment has been extended to low temperatures for the assessment of lipase activity at reduced temperatures (4, 8, and 15°C) in domestic wastewater [23]. Yet, methane production from LCFA mixtures has not been reported at a lipid content > 1% at low temperatures and warrants further investigation.

The inoculum source plays a crucial role in anaerobic treatment and more so at low temperatures, as the microbial community in the inoculum affects the substrate-degradation potential and the methanogenic activity [24, 25]. Both suspended and granular sludges have been used in low temperature anaerobic digestion (LTAD) studies to treat various wastewaters [13, 15, 22, 26–31]. Previous comparisons of granular to suspended sludges at low temperatures for methane production have contrasting conclusions, and their suitability appears to be case-specific. At 15°C, when Xing et al. [32] compared inocula from permafrost sites (lake sediment, pond silt, and wetlands) to mesophilic granular sludge, a higher methane production rate (71 mL-CH_4_/gVSS·d) from glucose of around 2 times was achieved using the waterfowl lake sediment (suspended sludge) compared to the other inocula. Conversely, at 15°C when Enright et al. [30] compared a sludge mixture (waste-activated sludge, cattle manure, and non-granular anaerobic sludge) to two granular sludges, more methane was produced by the granular sludges from acetate, H_2_/CO_2_, and ethanol.

In the anaerobic treatment of lipid-containing wastewater, both suspended sludge and granular sludge have been used as inoculum at mesophilic and thermophilic conditions. Granular sludge has been reported to be suitable for anaerobic LCFA treatment due to its lower specific surface area [33], a higher methanogenic activity (acetoclastic, hydrogenotrophic, propionate, and ethanol fermentation), and a lower oleate toxicity compared to suspended sludge while treating oleate (OLR 2-8 g-COD/L·d) [34, 35]. However, usage of suspended sludge as an inoculum source has been suggested for treating oleate-containing wastewater due to its higher LCFA sorption capacity [34, 35], as LCFA sorption is required for its degradation based on a sequential sorption-desorption mechanism [36]. Therefore, an evaluation of different inocula is needed for assessing the methane production from LCFA-containing wastewaters at low temperatures.

Apart from the physicochemical characteristics, the microbial community composition of the anaerobic sludge is affected by the high lipid or LCFA concentration and by the low temperature. LCFA degradation proceeds through the removal of 2 carbons in each *β*-oxidation cycle usually resulting in the production of acetate. In a microbial consortium, this *β*-oxidation requires the syntrophic coupling of acetogenic bacteria to the hydrogenotrophic methanogens to maintain low hydrogen partial pressures. The microbial communities involved in codigesting fat, oil, and grease (FOG) or LCFA at mesophilic and thermophilic conditions have been deciphered using high-throughput amplicon sequencing, and syntrophs have been concluded to also play a significant role in their degradation [37–40]. Only 7 acetogenic bacteria belonging to the families *Syntrophomonadaceae* (class Clostridia) and *Syntrophaceae* (class Deltaproteobacteria) [5, 41] are currently known to degrade LCFA, and the bacteria from the family *Clostridiaceae* (class Clostridia) have been suggested to degrade LCFA [42, 43], signifying the importance of bacterial taxa from these 3 families in LCFA degradation. Furthermore, the predominance of hydrogenotrophic methanogens has not been established at low temperatures in the anaerobic treatment of wastewaters with low-lipid and LCFA content [1, 16, 44–48] due to the thermodynamic preference for hydrogen utilization than acetate utilization at low temperatures [49]. An investigation of the microbial consortia involved in degrading the lipid- or LCFA-containing wastewaters at low temperatures through high-throughput amplicon sequencing has not yet been applied to the microbial consortia involved in degrading the lipid- or LCFA-containing wastewaters at low temperatures and could provide novel insights about the key taxa involved.

As the choice of inoculum for LTAD depends on its unique physicochemical characteristics and microbial community composition, the objective of this study was to assess the effect of the inoculum source on methane production from LCFA-containing synthetic dairy wastewater (SDW) in batch assays at low temperatures of 20 and 10°C. Amplicon sequencing was applied to characterize the microbial communities before and after the batch operation to study the changes in microbial community composition during incubation.

## 2. Materials and Methods

### 2.1. Inoculum and Substrate

Three inocula obtained from local sources were used in this study—two suspended sludges and one granular sludge. Suspended sludges from mesophilic anaerobic digesters of municipal wastewater treatment plants, Rahola (RD) and Viinikanlahti (VD), Tampere, Finland, were collected, sieved with a 16 mm mesh, and stored for 4 weeks under nitrogen-purged atmosphere at 7°C. Granular sludge (GS) was obtained from a mesophilic upflow anaerobic sludge blanket (UASB) reactor treating wastewaters from an integrated production of beta-amylase enzyme and ethanol from oat (Jokioinen, Finland) and stored for 3 weeks in nitrogen-purged atmosphere at 7°C. The sieved suspended and granular sludges were characterized (Table 1).

SDW and acetate were used as substrates in this study. SDW simulated constituents of dairy wastewater and contained a protein source (casein hydrolysate), a carbohydrate source (lactose monohydrate), and a fat source (LCFA mixture) (Table 2). The LCFA mixture consisted of palmitate, stearate, oleate, and linoleate in a COD ratio of 30 : 15 : 45 : 10 (Table 2) based on LCFA concentrations frequently found in dairy wastewaters [41, 50].

### 2.2. Methane Production in Batch Assays

Batch studies were performed using 120 mL serum bottles, with a liquid volume of 60 mL. In all assays, 15 mL of substrate stock solution (SDW or acetate) and 10-30 mL of inoculum (GS, RD, or VD) were added into the bottle, to ensure 2 g-COD/L of the substrate and 6 g-volatile solids (VS)/L of inoculum. 2 mL of stock nutrient solution was added to the bottles consisting of the following (g/L): MgSO_4_·7H_2_O (6), NH_4_Cl (16.8), KH_2_PO_4_·3H_2_O (0.24), Na_2_HPO_4_·2H_2_O (0.2), CaCl_2_·2H_2_0 (6), yeast extract (6), FeCl_2_·4H_2_O (0.12), H_3_BO_3_ (0.003), ZnCl_2_ (0.003), CuCl_2_·2H_2_O (0.002), MnCl_2_·4H_2_O (0.03), Na_2_MoO_4_·2H_2_O (0.001), CoCl_2_·6H_2_O (0.12), NiCl_2_·6H_2_O (0.005), Na_2_SeO_3_·5H_2_O (0.01), EDTA (0.06), and resazurin that was acidified with 1 mL of 36% hydrochloric acid prior to usage [51], and the volume was adjusted to 60 mL with distilled water. Subsequently, the pH in the assays was adjusted to 7.0 by adding 0.1 M sodium hydroxide or 0.1 M hydrochloric acid solutions. The headspace was flushed with nitrogen gas for 10 min and closed with a butyl rubber stopper to ensure anaerobic conditions after which it was sealed with aluminum crimp caps. The experiments were performed in triplicates and incubated at 20 or 10°C without shaking. Assays without substrates were prepared similarly, to act as blanks. Supernatant and sludge from the bottles were sampled at the end of the trial (200 d) for soluble COD (sCOD) and volatile fatty acid (VFA) measurements and for microbial community analysis, respectively. Assays prepared with the granular sludge as inocula will be referred to as GS, and the assays prepared with the municipal digester sludges—RD and VD—will be referred to as RD and VD, respectively, in the subsequent sections. A long assay duration (200 d) was undertaken due to the possibility for diauxic behavior as seen previously in the anaerobic treatment of LCFA-containing wastewater at 20°C in our study (data not published) and of fat-containing dairy effluent at 37°C [52].

### 2.3. Analytical Methods and Calculations

The biogas content (CH_4_, CO_2_, and H_2_) was analyzed using a Shimadzu GC-2010 gas chromatograph, equipped with a Porapak N column and a thermal conductivity detector. Helium was used as the mobile phase at a flow rate of 20 mL/min. Injector, oven, and detector temperatures were set at 110, 80, and 110°C, respectively. The volume of the gas was measured according to [53] and reported at standard temperature and pressure (STP). Liquid supernatant from the bottles at the end of 200 d was filtered through 0.45 *μ*m polyethersulphone syringe filters for sCOD and VFA measurements. For VFA measurement, a Shimadzu GC-2010 having ZB-Wax column was used for analysis, with helium as carrier gas at a flow rate of 19.6 mL/min. Injector and FID detector temperatures were both 250°C. The oven temperature was programmed to heat as follows: at 40°C for 2 min, then heated up to 160°C with 20°C/min and up to 220°C with 40°C/min, after which temperature was held at 220°C for 2 min.

The sCOD was measured using the potassium dichromate titrimetric method according to the Finnish standard SFS 5504. Total solids (TS), VS, total suspended solids (TSS), and volatile suspended solids (VSS) were measured according to APHA 2005 and alkalinity according to the Finnish standard SFS 3005. The concentrations of anions (SO_4_^2-^, PO_4_^3-^, and Cl^−^) in filtered samples were measured by a Dionex ICS-1600 Ion Chromatography System, equipped with an IonPac AS4A-SC anion exchange column, a DS6 heated conductivity cell, and 1.9 mM Na_2_CO_3_ and 1.7 mM NaHCO_3_ as the eluent at a flow rate of 1 mL/min.

Salinity measurements of the substrates were used for calculating dissolved gas (methane and carbon dioxide) concentrations and were carried out using a handheld conductivity meter WTW Cond 3210. Bunsen solubility coefficients were obtained using constant values from Yamamoto et al. [54] and were applied to estimate solubilized methane [55].

Methane production was determined by adding the methane dissolved in the liquid phase to the methane volume in the gaseous phase and subtracting the methane production in the assay blanks from this value. The methane yield (%) was calculated from the ratio of methane produced in each assay (mL/g-COD) to the theoretical methane volume produced per gram COD at standard temperature and pressure (STP) (350 mL/g-COD). Overall mass balances were performed in terms of COD at the beginning (Table 2) and at the end of the 200 d experiment for all the assays.

The cumulative methane production from the assays was fitted with the modified Gompertz equation:

(1) $$\begin{array}{c} M\left(t\right)=P\cdot \exp\left[-\exp\left\{\frac{R_{\text{m}}\cdot e}{P}\left(\lambda -t\right)+1\right\}\right], \end{array}$$

where *M*(*t*) is the cumulative methane production (mL-CH_4_) at time *t* (d), *P* is the methane production potential (mL-CH_4_ or mL-CH_4_/gVS), *R*_m_ is the maximum methane production rate (mL-CH_4_/d), and *λ* is the lag phase for methane production (d). The curve fitting tool in MATLAB R2017b was used to calculate the lag phase (*λ*) and the maximum methane production rate (*R*_m_).

### 2.4. DNA Extraction, Quantification, and 16S rRNA Amplicon Sequencing

The microbial samples (three inocula and from all the assays after 200-day incubations) were stored at -20°C immediately upon sampling. After thawing the samples at 4°C, the total DNA was coextracted along with RNA from the microbial samples [56]. The concentration of extracted DNA was quantified with a Qubit Fluorometer (Life Technologies), and the DNA purity was measured using a NanoDrop spectrophotometer (NanoDrop Technologies, Wilmington, USA). The DNA samples were sent to FISABIO (Spain) for PCR amplification of the V4 region of the 16S rRNA gene with universal primers 515f and 806r [57] and amplicon sequencing with the Illumina MiSeq platform.

### 2.5. Bioinformatics and Statistical Tools

Computational analysis of the sequenced data was done using the Quantitative Insights Into Microbial Ecology (QIIME v1.9) pipeline [58]. The average length of the forward and reverse reads was 291 and 294 bp, respectively. The paired-end reads were joined using a fastq-join method [59] with a min overlap of 50 bp and a perc_max_diff of 15%, after which quality filtering was performed using the split_libraries_fastq.py script in QIIME [58]. The sequences were clustered into operational taxonomic units (OTUs) using the open-reference OTU picking with the BLAST method, and taxonomy assignment was performed with the Silva 128 consensus taxonomy at all levels [60, 61]. Chimeric sequences were identified using ChimeraSlayer, and the final OTU table was generated from the nonchimeric sequences using the script make_otu_table.py.

The dataset consisted of 5743805 sequences in total, which clustered into 12326 OTUs at 97% similarity level. There were 127 abundant OTUs that formed 99.9% of the community which were subsampled to an even sequencing depth of 26511 (based on the number of reads in the smallest sample) for alpha diversity metrics (diversity within samples). Community diversity and species richness were estimated using Chao1 and Shannon indices and the number of observed OTUs. The alpha diversity indices for the inoculum duplicates and assay triplicates are presented as the averaged values of the microbial community composition within the inoculum duplicates or within the experimental assay triplicates after 200 days.

The subsampled data set consisting of sequences representing the 127 most abundant OTUs was used in subsequent analyses. The subsampled OTU table was fourth-root-transformed for even distribution, and a resemblance matrix was constructed using Bray-Curtis similarity in the Plymouth Routines In Multivariate Ecological Research (PRIMER) V7 [62]. Cluster analysis was performed to discern patterns in the microbial community of the different inocula in the low temperature treatment of LCFA-containing wastewater. Cluster analysis through hierarchical clustering (group average method) was performed at the taxonomic class level on the operational taxonomic units (OTUs) and separately on the assay samples to plot the dendrograms. The fourth-root-transformed OTU table was used for representing the microbial community composition in a shade plot with dendrograms.

## 3. Results and Discussion

### 3.1. Inoculum Characteristics and Microbial Community Composition

#### 3.1.1. Physicochemical Characteristics

Among the three mesophilic inocula, the granular sludge (GS) had a higher VS : TS ratio (0.86) compared to the municipal digester sludges—RD and VD (0.55)—while GS had lower sCOD than the municipal digester sludges (520 vs. 1100-2500 mg/L) (Table 1). GS contained some VFA (147 mg/L) whereas the VFA was lower than the detection limit in both of the municipal digester sludges. The two municipal digester sludges differed in several properties; e.g., the TS, sCOD, and PO_4_^3-^ concentrations were several-fold different (Table 1) suggesting the impact of inflow wastewater characteristics and plant operation, as the two municipal wastewater treatment facilities have similar unit processes, which are the primary sedimentation and activated sludge processes, followed by mesophilic anaerobic digestion of the excess sludge generated.

#### 3.1.2. Microbial Community Composition of the Inocula

High-throughput amplicon sequencing was used to investigate the microbial community composition of the three inocula. The municipal digester sludges (RD and VD) had a higher microbial community diversity (Chao1: 104-108, Shannon: 4.53-4.73, observed_otus: 103-105) compared to the GS inoculum (Chao1: 93, Shannon: 3.69, observed_otus: 79) (Table 3), although the three inocula were obtained from mesophilic reactors. The bacterial classes *Anaerolineae* (relative abundance 7.4-23.25%), *Clostridia* (relative abundance 0.1-0.6%), and *Synergistia* (relative abundance 6.3-10.6%) and the archaeal class *Methanomicrobia* (relative abundance 7.8-19%) were present in all the three inocula. Although the municipal digester sludges (RD and VD) had a similar microbial community composition, the relative abundance of *Methanomicrobia* (13 vs. 7%) and *Anaerolineae* (23 vs. 13%) was higher in RD than in VD inoculum. Overall, GS inoculum had a high relative abundance of *Methanobacteria* (21.5%), uncultured *Aminicenantes* (25%), and *Deltaproteobacteria* (5%) compared to that of the municipal digester sludges.

The microbial community composition and diversity in the three inocula were different. The higher microbial community diversity in the municipal digester sludges (Table 3) was likely due to the wide variety of substrates received by the municipal wastewater treatment plants. In comparison, GS was sourced from an UASB treating carbohydrate and alcohol-based wastewater, which potentially narrowed the diversity of the microbial community.

To date, only 7 species from the classes *Clostridia* or *Deltaproteobacteria* are known to degrade LCFA (carbon atoms > 12) of which only 4 species (*Syntrophomonas sapovorans*, *Syntrophomonas curvata*, *Syntrophomonas zehnderi*, and *Thermosyntropha lipolytica*) from the class *Clostridia* are currently known to degrade the unsaturated LCFAs, e.g., oleate and linoleate [5], and have also been found in various LCFA-fed digesters [63–66]. Moreover, at low temperatures, the Deltaproteobacterial class plays a significant role along with the archaeal classes of *Methanobacteria* and *Methanomicrobia* [22, 44, 45, 47, 67, 68]. Therefore, monitoring the bacterial and the archaeal taxa belonging to the classes *Deltaproteobacteria* and *Clostridia*, and *Methanobacteria* and *Methanomicrobia*, respectively, was considered of special interest while investigating anaerobic LCFA treatment at low temperatures in this study.

### 3.2. Methane Production at Low Temperature from SDW and Acetate

**Figure 1:**
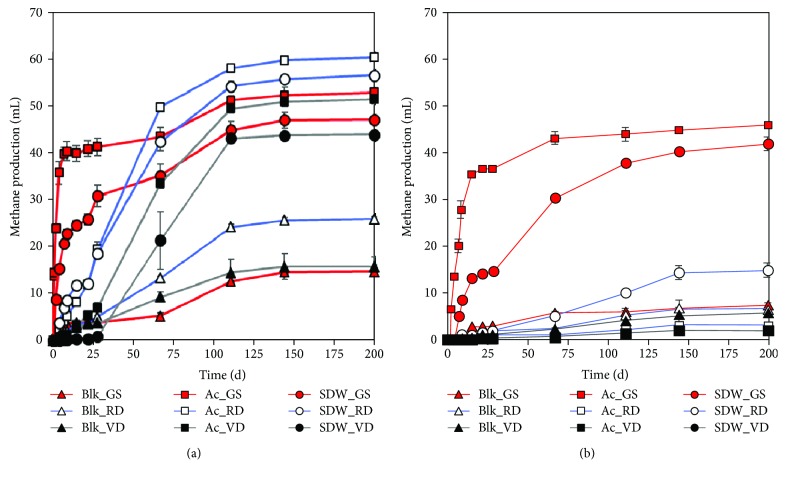
Methane production (mL) at (a) 20°C and (b) 10°C from different inocula (GS: granular sludge; RD: Rahola Digestate; VD: Viinikanlahti Digestate) incubated with no substrate (Blk), acetate (Ac), and synthetic dairy wastewater (SDW).

The potential of the three different inocula for methane production from SDW and acetate was studied in batch assays at 10 and 20°C (Figure 1). Blank assays without any added substrates were prepared to subtract the methane production from their corresponding assays with the added substrates. The cumulative methane production curves were fitted with the Gompertz equation (*R*-square: 0.9-0.99) and were used for calculating the lag time and the maximum methane production rate (*R*_m_).

Granular sludge (GS) inoculum started methane production rapidly both at 20°C (0.6 d) and 10°C (1.5 days) with both of the substrates while the other two inocula (VD, RD) had a longer and comparable (20-23 d) lag phase (*λ*) at 10°C with acetate and SDW. However, at 20°C, VD had several-fold longer lag phases than RD (18-50 d vs. 4-9 d, respectively) (Figure 1, Table 4). At 20°C, the *R*_m_ was comparable or higher with GS than the municipal digester sludges with SDW (1.6 and 0.9) and acetate (9.5 and 1). At 10°C with SDW, the GS had 4.7 times higher *R*_m_ than the RD, while other RD and VD assays at 10°C produced little methane (Table 4). Methane yields within 10 days were substantially higher with the granular sludge—86 and 65% with acetate and 45 and 20% with SDW at 20 and 10°C, respectively (Figure 1), compared to the municipal digester sludges (methane yields less than 20%). After 200 days, methane yield with granular sludge was overall comparable at 20 and 10°C from acetate (83-95% and 91-95%, respectively) or SDW (70-82% and 79-85%, respectively) (Table 4). Contrastingly, methane yields from acetate and SDW with the municipal digester sludges (RD and VD) were much higher at 20°C (75-93% and 60-75%, respectively) compared to 10°C (<20%). The results show that the municipal digester sludges were strongly affected by temperature. Methane was produced with RD at 10°C with SDW but not with acetate which is a simpler methane precursor than SDW.

**Figure 2:**
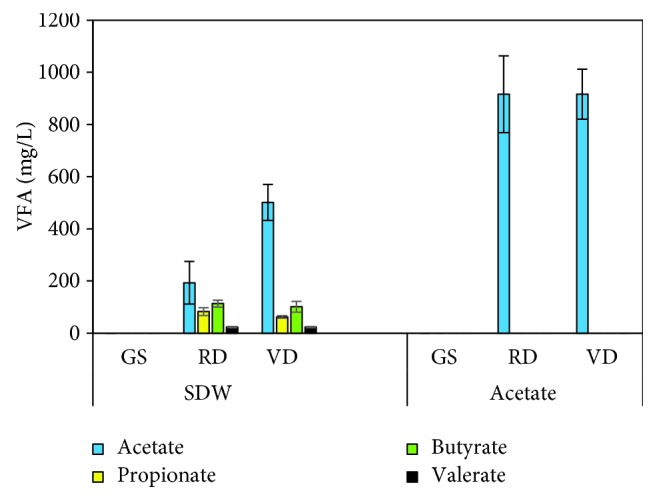
Residual volatile fatty acid (VFA) concentration at 10°C with synthetic dairy wastewater (SDW) and acetate at the end of the 200 d experiment (GS: granular sludge; RD: Rahola Digestate; VD: Viinikanlahti digestate).

**Figure 3:**
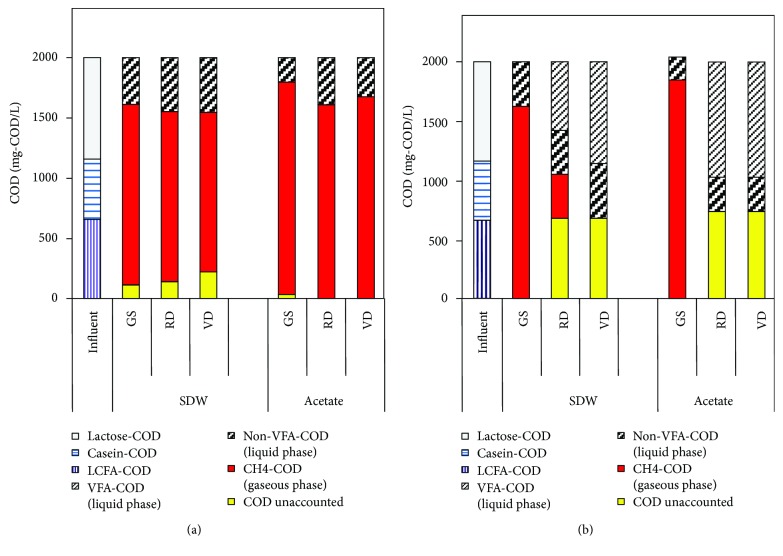
Mass balance of COD at (a) 20°C and (b) 10°C in the assays with different inocula (GS: granular sludge; RD: Rahola Digestate; VD: Viinikanlahti digestate) and with the substrate synthetic dairy wastewater (SDW) or acetate.

sCOD removal at 20°C was similar with all three inocula, 78-81% with SDW and 81-90% with acetate (Fig.  S1), but at 10°C, a higher sCOD removal was obtained with GS (82% in SDW-addition and 91% in acetate-addition) than with the municipal digester sludges. Despite the sCOD removal of 29-53% in the municipal digester sludges at 10°C (Fig.  S1), methane production was low (methane yield < 20%), which suggests that the sCOD removal was apparently due to the sorption by inoculum biomass [36]. Moreover, after 200 days, VFAs were detected only with the municipal digester sludges at 10°C (total VFAs of 290-780 mg/L with SDW and 760-1070 mg/L with acetate). With SDW, the most abundant VFA was acetate (110-580 mg/L), and in the acetate-fed assays, only acetate (760-1070 mg/L) was found (Figure 2), which suggests an inhibition of acetotrophic activity. COD balance (Figure 3) shows that 9-19% and 19-23% of the sCOD (non-VFA) remained in the acetate and SDW assays, respectively, after the 200 d period. This non-VFA sCOD might have been produced due to the release of endogenous decay products. The unaccounted COD in the acetate and SDW assays increased at 10°C compared to 20°C (Figure 3) likely due to cell growth or substrate sorption to the biomass. The VFA accumulation did not affect the pH and was 6.9-7.1 in all the assays at 20 and 10°C at the end of the experiment.

The methane production from acetate or SDW with GS at 20 and 10°C indicates that the COD from the carbohydrate (lactose), protein (casein), and LCFA (saturated and unsaturated) fractions was metabolized by the anaerobic consortia in GS (Figure 3). On the contrary, the decrease in temperature diminished methanogenesis in RD and VD assays. Moreover, at 10°C, the fraction of SDW hydrolyzed (sum of the VFA-COD and the CH_4_-COD in Figure 3) was <50%, indicating that the hydrolysis and acidification of carbohydrate and protein fractions were also diminished at 10°C. Even in the acetate-fed assays at 10°C, less than 50% of the substrate uptake was detected in RD and VD assays. The presence of high hydrogen partial pressures limits the syntrophic LCFA degradation by *β*-oxidation, and LCFA further inhibits the trophic groups in anaerobic microbial consortia, which could have affected the SDW uptake at 10°C. However, substrate uptake (acetate and SDW) at 10°C was not energetically limited by hydrogen accumulation/increased hydrogen partial pressure in RD and VD, as hydrogen was not found in the gas phase. Therefore, the lack of substrate uptake was related to the inhibition in acetotrophic activity in the digestate inocula at 10°C.

Previously, single LCFAs—linoleic acid (30 mg/L), oleic acid (30 mg/L), and stearic acid (10 mg/L)—have been reported to inhibit methanogenesis from 100 mg/L of acetate at 21°C [18, 19]. In this study, however, a combination of LCFAs (34.7 mg/L of linoleate, 91.2 mg/L of oleate, and 33.8 mg/L of stearate) present in SDW was converted to methane by GS at 20 and 10°C and by the two municipal digester sludges at 20°C (Figure 1). In spite of a higher LCFA load in the current experiment (38 mg LCFA/g·VS) than the inhibitory concentration reported elsewhere with a single LCFA (20 mg LCFA/g·VS and 6.67 mg LCFA/g·VS at 21°C [18, 19]), methane was produced from a mixture of saturated and unsaturated LCFAs in this study. To the best of our knowledge, this is the first report of methane production from a LCFA mixture containing unsaturated LCFAs (oleate and linoleate) at 10°C, wherein the methane production was driven by the inoculum origin. These results can be used for understanding and developing anaerobic processes for the low-temperature treatment and methane production from lipid-containing wastewaters.

### 3.3. Effect of Low Temperature and SDW on Microbial Community Composition

#### 3.3.1. Microbial Community Diversity after 200 Days

Due to the differences in the relative abundance of the microbial community composition in the three inocula, and the differing methane production in the assays at different temperatures and substrates, an underlying shift in the bacterial and archaeal taxa was envisaged after the 200 d incubation period. High-throughput amplicon sequencing was used to investigate the microbial community composition in the assays after 200 days. A total of 12326 OTUs were found, of which 99.9% of the microbial community belonged to 127 OTUs consisting of 33 bacterial and 4 archaeal classes. Therefore, a small portion of the taxa (0.01% of total OTUs) comprised a 99.9% portion of the microbial community. Moreover, similar to their inoculum, even after the 200 d, the municipal digester sludges (RD and VD) had a higher microbial diversity (Chao1: 106-116, Shannon: 4.31-5.13) compared to the GS (Chao1: 93, Shannon: 3.69, observed_otus: 79) (Table 3). Based on the Chao1 and the number of observed OTUs, the community diversity increased in VD and RD but decreased in GS during the 200-day period, independent of the temperature or the substrate.

#### 3.3.2. Relative Abundance of the Archaeal Classes after 200 Days

**Figure 4:**
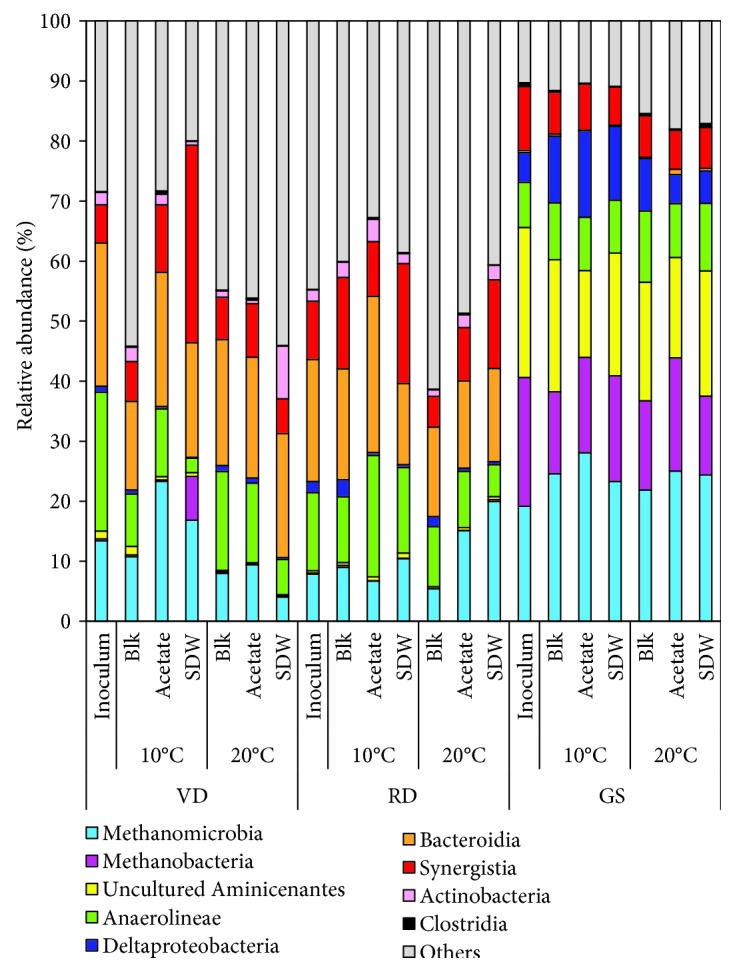
Relative abundance (%) of the bacterial and archaeal classes found in the 16S rRNA amplicon libraries in the samples during the 200 d experimental period from different inocula (GS: granular sludge; RD: Rahola Digestate; VD: Viinikanlahti Digestate) incubated at 10°C and 20°C with no substrate (Blk), acetate, and synthetic dairy wastewater (SDW). Detailed information on the sample names is shown in Table S1.

**Figure 5:**
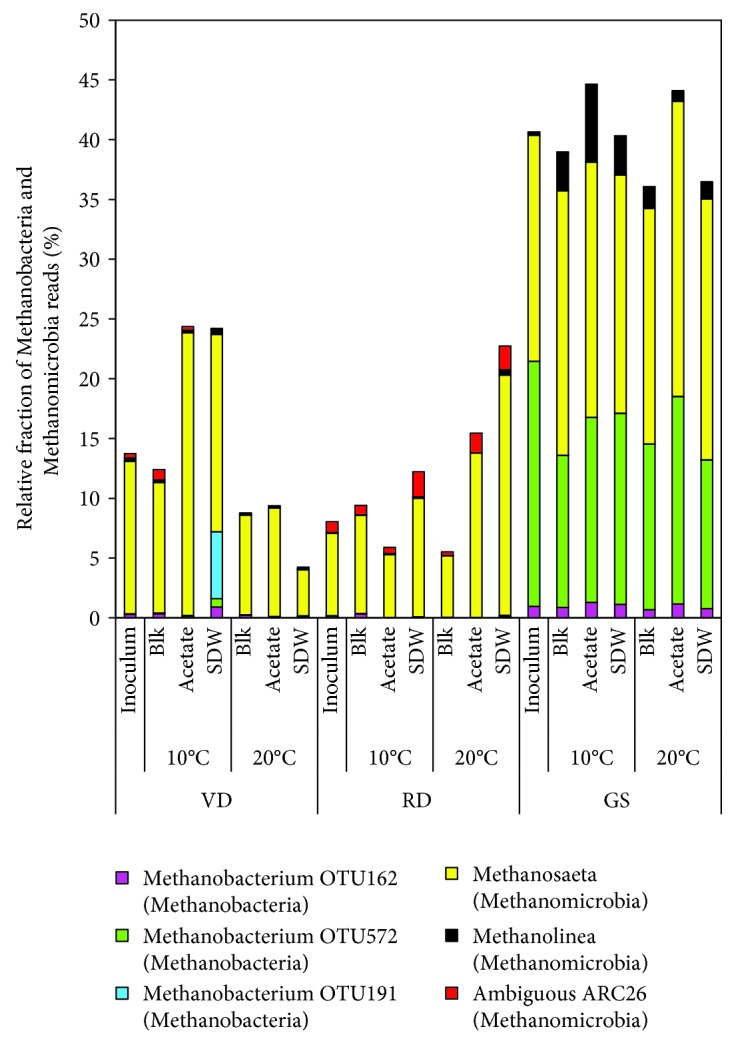
Relative fraction (%) of the archaeal genera belonging to the classes Methanobacteria and Methanomicrobia found in the 16S rRNA amplicon libraries in the samples during the 200 d experimental period from different inocula (GS: granular sludge; RD: Rahola Digestate; VD: Viinikanlahti Digestate) incubated at 10°C and 20°C with no substrate (Blk), acetate, and synthetic dairy wastewater (SDW). Classes are mentioned within brackets. Detailed information on the sample names is shown in Table S1.

The bacterial and archaeal taxa that changed with the substrate and temperature after the 200 d incubation period were assessed by their change in relative abundance. Only the OTUs with relative abundance above 0.1% are further discussed. In all the assays, 9 archaeal OTUs were found belonging to *Methanobacteria* (3 OTUs), *Methanomicrobia* (3 OTUs), *Thermoplasmata* (1 OTU), and WCHA1-57 (2 OTUs). The class *Methanobacteria* was found at a higher relative abundance in the GS assays (13-19%) compared to VD and RD (less than 7%) (Figure 4), but its relative abundance decreased over the incubation period compared to the GS inoculum (21%) bearing no temperature-specific or substrate-specific trends. The class *Methanomicrobia* was found in all the assays after the 200 d incubation, with a higher relative abundance in GS (22-28%, increased from 19%) compared to VD (increased from 13% to 19-23% at 10°C) and RD (increased from 8% to 15-20% at 20°C) (Figure 4). Within the class *Methanomicrobia*, the relative abundance of the hydrogenotrophic *Methanolinea* increased in the GS (0.8-6.5% vs. 0.3% in inoculum), while the hydrogenotrophic ARC26 (ambiguous taxon) had a higher relative abundance in VD and RD (0.1-2%) than in GS (Figure 5). *Methanosaeta* was the only acetoclastic archaeal genus found after the 200 days, and its relative abundance increased at 20°C in the acetate-fed assays compared to blank assays in GS (from 20 to 25%), VD (from 8.3 to 9.1%), and RD (from 5.1 to 13.8%). At 10°C, in the acetate-fed assays, the relative abundance of *Methanosaeta* remained high in GS (20-22%) and increased only in VD (from 11 to 24%) (Figure 5). The members of the class *Methanobacteria* and *Methanomicrobia* have previously been detected in psychrophilic environments [69] and in anaerobic LCFA degradation assays [70] and were also found to be prevalent in this study.

VFA accumulation (predominantly acetate) was observed with RD and VD assays at 10°C when fed with SDW. In addition, in the RD and VD assays at 10°C fed with acetate, only 50% of acetate was consumed with negligible methane production (Figure 3). Even with a high relative abundance of *Methanosaeta* (only acetoclastic taxa found in this study) in VD and of hydrogenotrophic taxa in VD and RD at 10°C, negligible methane was produced in RD and VD assays at 10°C. This inhibition of acetotrophic activity at 10°C in RD and VD assays suggests acetate uptake by syntrophic acetate-oxidizing bacteria (SAOB). SAOB growth can be energetically feasible at lower temperatures close to the thermodynamic equilibrium, and their presence has been confirmed at temperatures as low as 7°C [71–73]. SAOB are slow-growing and have been isolated in the presence of strong selection pressures [74, 75]. In our study, the presence of stressors, such as low temperature, likely imparted a competitive advantage to the acetate oxidizers for acetate uptake compared to the acetoclastic methanogen, *Methanosaeta*. This advantage is conferred to the acetate oxidizers from syntrophic coupling with hydrogenotrophic methanogens, due to more favorable energetics of the hydrogenotrophic methanogenic pathway at lower temperatures (10°C). A shift in the methanogenic pathway from acetotrophic to hydrogenotrophic has been observed previously with a temperature drop [1, 47, 48], and LCFA presence has been known to inhibit acetoclastic methanogens more than hydrogenotrophic methanogens [5, 7], thereby suggesting a need for maintaining high hydrogenotrophic activity for methane production at low temperatures. In contrast to the previous studies at low temperatures, this study highlights the need for maintaining high acetotrophic activity for LCFA utilization at low temperatures through methanogenic archaea and/or syntrophic acetate oxidation bacteria (SAOB) (further discussed in Section 3.3.3).

#### 3.3.3. Relative Abundance of the Bacterial Classes after 200 Days

In all the assays, the bacterial classes *Anaerolineae* (relative abundance 2.4-20%), *Clostridia* (0.1-0.7%), and *Synergistia* (6-33%) were found at the end of the batch incubation. The classes *Bacteroidia* and *Actinobacteria* were present in higher abundance in both municipal digester sludges than in GS (*Bacteroidia* 15-26% vs. <1%, respectively, and *Actinobacteria* 1-9% vs. <0.1%, respectively), while GS had a higher relative abundance of uncultured *Aminicenantes* (15-22% vs. 0.2-1.4%) and *Deltaproteobacteria* (5-14% vs. 0.2-2.8%) (Figure 4). The large variation in relative abundance of different classes is due to the changes in temperature and substrate and are discussed below.

**Figure 6:**
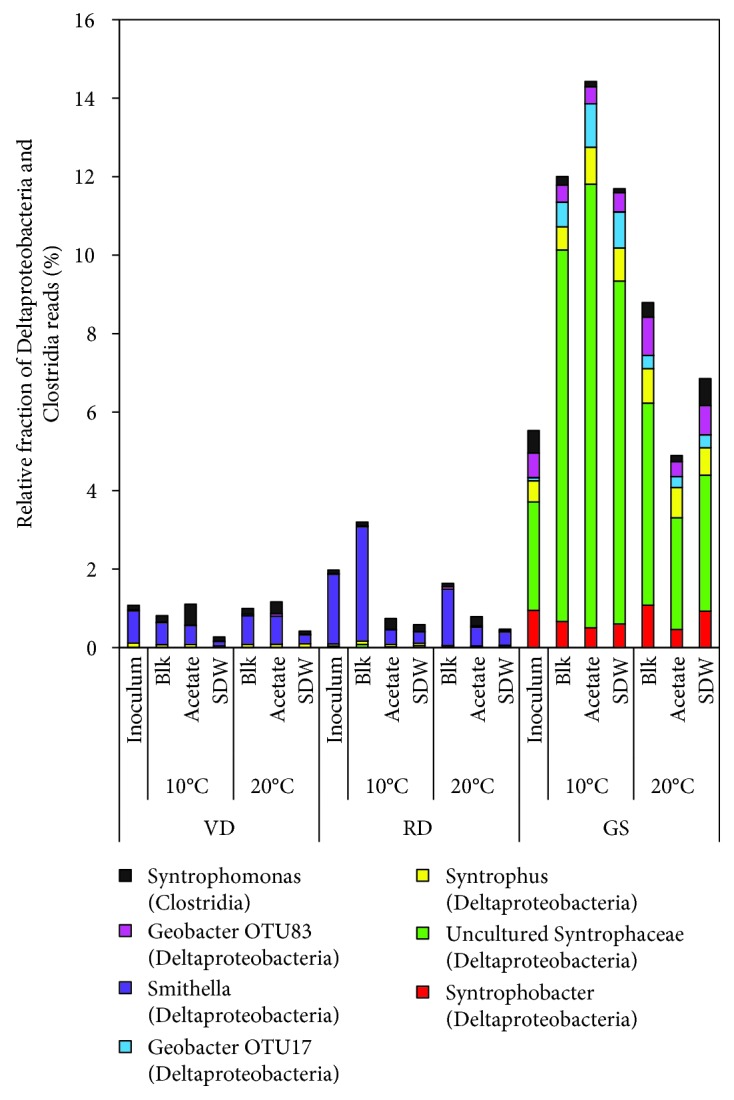
Relative fraction (%) of the bacterial genera belonging to the classes Deltaproteobacteria and Clostridia found in the 16S rRNA amplicon libraries in the samples during the 200 d experimental period from different inocula (GS: granular sludge; RD: Rahola Digestate; VD: Viinikanlahti Digestate) incubated at 10°C and 20°C with no substrate (Blk), acetate, and synthetic dairy wastewater (SDW). Classes are mentioned within brackets. Detailed information on the sample names is shown in Table S1.

After the 200 d incubation period, 6 OTUs were found in the class *Deltaproteobacteria*, among which 4 belonged to the order *Syntrophobacterales* (families *Syntrophaecea*e (3 OTUs), *Syntrophobacteraceae* (1 OTU)) and two to the order *Desulfuromonadales* (family *Geobacteraceae*) (Figure 6). The relative abundance of the acetogenic bacterium *Syntrophus*and of an uncultured taxon (from family *Syntrophaceae*) increased in the GS at 10°C (from 3.3% to 9.6-12.3%) during the 200 d incubation (Figure 6), highlighting the role of the acetogenic bacteria from the family *Syntrophaceae* in LCFA degradation at 10°C. The members of the family *Syntrophaceae* are known to degrade the saturated LCFAs—palmitate (C16:0) [65], stearate (C18:0), and heptanoate (C17:0)—at mesophilic conditions [66], and only one known species from the family *Syntrophaceae*—*Syntrophus aciditrophicus*—has been found to degrade two saturated LCFAs (palmitate (C16:0) and stearate (C18:0)) [76]. *Syntrophus aciditrophicus* has a growth range at temperatures of 25-42°C; thus, a psychrotolerant growth mode at 10°C of *Syntrophus-*like taxa for metabolizing the unsaturated LCFAs (oleate and linoleate) is found in this study.

Furthermore, at 10°C, the GS had a methane yield of 82% from the substrate SDW (Table 2) that constituted of 33% LCFAs (18% unsaturated LCFAs (C18:2, C18:1) and 15% saturated LCFAs (C16:0, C18:0)). Therefore, this study confirms the possibility of methane production from the saturated and unsaturated C16 and C18 acids at low temperatures (10 and 20°C) with a crucial role played by syntrophs and acetotrophs. While the metabolic pathways involved in the degradation of saturated and unsaturated LCFAs individually and in mixture at low temperatures are not known yet, they should be elucidated with further studies.

Another important class *Synergistia* (16 OTUs) was found in all the samples (6.3-10.6%) (Figure 4). Although the precise function of *Synergistia* in degrading LCFA remains unconfirmed, it has been found in the core microbiome of mesophilic and thermophilic LCFA-fed digesters [70]. Of the 16 OTUs found in this study in the class *Synergistia*, 5 belonged to the thermophilic or mesophilic amino acid-degrading genera *Thermovirga* [77], *Lactivibrio* [78], or *Aminivibrio* [79]. As casein constituted 25% COD in the SDW, the presence of amino-acid degraders is expected; however, few of the other 11 *Synergistia* taxa could have a role in methane production from SDW.

**Figure 7:**
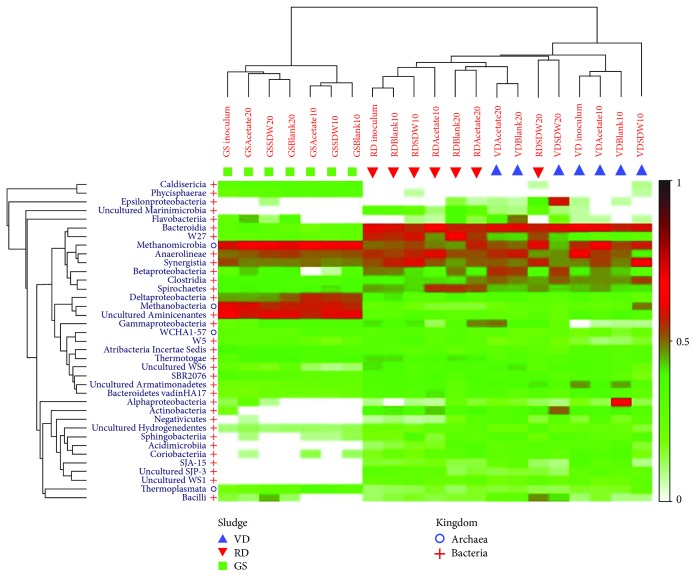
Heat map showing the clustering and relative abundance of the bacterial and archaeal classes that formed 99.9% of the microbial community in the assays inoculated with VD, RD, and GS and fed with no substrate (blank), acetate, or synthetic dairy wastewater (SDW) at 10°C and 20°C. Symbols + and o indicate the kingdom bacteria and archaea, respectively, on the y-axis. Symbols ▲, ▼, and ■ represent the inoculum used in the assays as VD, RD, and GS, respectively, on the x-axis. Detailed information on the sample names is shown in Table S1.

In GS, *Aminicenantes* clustered closely with the hydrogenotrophic *Methanobacteriales* (Figure 7). Additionally, *Synergistetes* was found in a high relative abundance in RD and VD assays, and a recently found thermophilic SAOB, *Gelria* (order *Thermoanaerobacterales*) [80], was found in a GS assay when fed with SDW at 20°C. The taxa from *Aminicenantes*, *Synergistetes*, and uncultured *Gelria* are putative SAOBs in this study due to their capacity and known role in syntrophic acetate oxidation [73, 80, 81]. However, as these species are uncultured, their specific functions cannot be confirmed. While syntrophic electron transfer by *Aminicenantes* has been suggested previously in a stratified lake at a low temperature of 7°C [73], the *Synergistetes* have been shown to perform syntrophic acetate oxidation with *Methanoculleus* and *Methanosarcina* in mesophilic anaerobic reactors [81]. Moreover, *Geobacter* (2 OTUs) were also enriched in GS assays at 10°C with concurrent high relative abundance of *Methanosaeta* in acetate and SDW assays. As *Geobacter* species can facilitate syntrophic electron transfer with *Methanosaeta* [82], there is also a possibility for *Geobacter*-mediated syntrophic acetate oxidation in this study. Furthermore, *Smithella* was found in the municipal digester sludges but not in GS and could have played a role in the LCFA degradation in SDW at 20°C due its known role in syntrophic acetate oxidation at 22°C [83]. As the number of SAOB candidates has been increasing recently and many SAOB taxa remain unknown, the possibility of SAOB at lower temperatures in this study cannot be excluded. Advanced molecular techniques, such as metagenomics, along with quantitative methods like qPCR are required to evaluate the activity and functional role of *Syntrophus* and *Syntrophus-*like taxa in degrading the unsaturated and saturated LCFAs (palmitate, stearate, oleate, and linoleate) and for the identification of putative SAOB for syntrophic interspecies electron transfer at low temperatures of 20°C and 10°C.

#### 3.3.4. Patterns in Microbial Community

The clustering of the microbial samples from different assays after 200 d was represented through dendrograms on a shade plot and principal coordinate analysis (PCoA) to discern patterns in the microbial community (Figures 7 and S2). The PCoA analysis revealed that the first two axes explained approximately 80% (first axis 72.5% and second axis 9%) of the variation in the microbial community present in the VD, RD, and GS sludges (Fig.  S2). Both the dendrograms in the shade plot and the similarity % in the PCoA plot show that the microbial community compositions in RD and in VD were similar to each other and clustered closely at 70% similarity in the blank and acetate, and SDW-fed assays. But the microbial community compositions of assays (blank, acetate, or SDW-fed) with GS inoculum had a higher similarity among themselves (85%) and clustered further from the RD and VD (Fig.  S2), to which the GS shared a lower similarity (66% with RD and VD) (Figure 7).

This clustering indicates that even after a prolonged incubation time of 200 days at a specific selection pressure (of temperature and substrate), the composition of the microbial communities did not converge. The only instance where an overlap in microbial classes was found between assays of different inoculum types was with the acetate-fed municipal digester sludges at 20°C (Figures 7 and S2). This suggests the effect of acetate in converging the microbial communities in VD and RD, although even the initial microbial community composition of VD and RD was similar. A continuous reactor operation may further reveal the development of microbial community assembly occurring under strong selection pressures in various inocula considering their dissimilar microbial composition. The similar performance (methane production) obtained with acetate or SDW with the three inocula at 20°C, irrespective of the differences in initial microbial community composition, suggests that functions were conserved in VD, RD, and GS at 20°C. However, this functional conservation was not effective at 10°C due to the strong abrupt selection pressures that hindered the metabolic functions and thus the methane production at 10°C compared to 20°C.

The presence of syntrophic partners (acetogenic bacteria with methanogenic archaea) is crucial for the LCFA degradation and was evaluated through their cooccurrence through the dendrograms. The OTU clustering showed that the bacterial classes *Synergistia* and *Anaerolineae* grouped with the archaeal class *Methanomicrobia* (the cluster was present in all samples) and conferred functional conservation to VD, RD, and GS at 20°C. Additionally, in GS, the bacterial classes *Deltaproteobacteria* and uncultured *Aminicenantes* grouped with the archaeal class *Methanobacteria* (the cluster present only in GS) (Figure 7), which is indicative of functional redundancy in GS that was not present in the RD and VD. As optimal metabolite transfer is aided by close proximity between syntrophic partners [84, 85], the formation of the distinct clusters in this study (Figure 7) signifies structural proximity at a molecular level and indicates an underlying interactive functional role and putative ecological niche associations. Previously, Grabowski et al. [66] had demonstrated the formation of close spatial associations of acetogens from *Deltaproteobacteria* (*Syntrophus-*related) with the methanogenic archaea—*Methanocalculus* and *Methanosaeta*—using in situ hybridization. In our study, the close clustering of *Deltaproteobacteria* and *Methanobacteria* in the GS could have facilitated the degradative capacity and the methane production with GS. An investigation using high-throughput sequencing (metagenomics) of closely clustered taxa involving bacterial acetogens and methanogenic archaea (such as *Methanomicrobia* with *Synergistia* and *Methanobacteria* with *Deltaproteobacteria* in this study) could confirm the functional role of the syntrophic partners involved in the clusters.

## 4. Conclusions

For the first time, the anaerobic conversion of mixed LCFA (saturated as well as mono- and polyunsaturated) containing wastewater (SDW) to methane is demonstrated at low temperatures of 20 and 10°C. High-throughput amplicon sequencing revealed the crucial roles of the acetotrophic activity by *Methanosaeta* and putative SAOB and of the psychrotolerant bacteria from the family *Syntrophaceae* (*Syntrophus* and uncultured taxa) in LCFA degradation at 10°C. Unacclimated granular sludge achieved high methane yields (70-85%) with SDW and was found to be a suitable inoculum for the treatment of mixed LCFA containing wastewater at both 20 and 10°C. Unacclimated municipal digester sludges can be employed for treating the mixed-LCFA containing wastewaters at 20°C but not at lower temperatures. This study provides the basis for the inoculum selection by the evaluation of acetotrophic activity and the initial microbial community characteristics for producing methane from mixed LCFA-containing wastewater at low temperatures (up to 10°C).

## Figures and Tables

**Table 1:** Characteristics of the three inocula used in the assays at 10 and 20°C.

Parameters	Granular sludge (GS)	Rahola Digestate (RD)	Viinikanlahti digestate (VD)
Soluble COD (mg/L)	525 ± 10	2520 ± 95	1080 ± 40
Volatile fatty acids (mg/L)	147 ± 2.0	—^∗^	—^∗^
pH	6.8 ± 0.2	7.3 ± 0.1	6.9 ± 0.1
Alkalinity (mM)	44.0 ± 0.1	78.0 ± 0.1	94.0 ± 0.1
TS (g/L)	42.0 ± 5.0	51.0 ± 1.5	20.1 ± 1.0
VS (g/L)	36.0 ± 4.0	28.0 ± 1.0	11.1 ± 0.2
TSS (g/L)	39.0 ± 2.0	51.0 ± 2.0	20.0 ± 0.2
VSS (g/L)	34.0 ± 1.6	27.0 ± 0.15	11.0 ± 0.2
SO_4_^2-^ (mg/L)	4.4 ± 0.01	184.0 ± 0.8	67.0 ± 0.1
PO_4_^3-^ (mg/L)	6.6 ± 0.01	57.0 ± 0.3	1.5 ± 0.03
Cl^−^ (mg/L)	17.0 ± 0.1	9.0 ± 0.01	29.0 ± 0.05

^∗^Below detection limits.

**Table 2:** Composition of the synthetic dairy wastewater (SDW) used as a substrate in the assays with VD, RD, and GS at 10 and 20°C.

Substrate component	LCFA	% of COD	Concentration (mg/L)
Casein		25	348
Lactose		42	730
LCFA, total		33	229
	Palmitate	10	69
	Stearate	4.9	34
	Oleate	13.2	91
	Linoleate	4.9	35

**Table 3:** Alpha diversity metrics (diversity within samples)—Chao 1 indices, Shannon indices, and number of observed OTUs for all the OTUs achieving 99.9% cut-off in the overall relative abundance. Detailed information on the sample names is shown in Table S1.

	Chao1	Shannon	observed_otus
VD inoculum	108 (1)	4.73 (0.05)	105 (0.5)
VD_Blank_10	106 (4)	4.38 (0.1)	104 (1.2)
VD_Acetate_10	108 (3)	4.62 (0.07)	106 (1.4)
VD_SDW_10	109 (3)	4.31 (0.04)	105 (1.2)
VD_Blank_20	116 (12)	5.13 (0.01)	109 (2.1)
VD_Acetate_20	115 (8)	5.06 (0.05)	109 (1.7)
VD_SDW_20	115 (4)	4.79 (0.33)	109 (0.8)
RD inoculum	104 (0)	4.53 (0.14)	103 (0.5)
RD_Blank_10	108 (3)	4.76 (0.1)	105 (2.9)
RD_Acetate_10	113 (0)	4.49 (0.02)	107 (0.5)
RD_SDW_10	114 (9)	4.61 (0.09)	104 (2.4)
RD_Blank_20	112 (1)	4.46 (0.02)	110 (0.1)
RD_Acetate_20	113 (1)	5.12 (0.04)	111 (0.9)
RD_SDW_20	111 (4)	4.8 (0.2)	109 (5)
GS inoculum	93 (14)	3.69 (0.01)	79 (2.5)
GS_Blank_10	82 (16)	3.8 (0.13)	70 (3.7)
GS_Acetate_10	77 (3)	3.86 (0.02)	66 (2.2)
GS_SDW_10	80 (11)	3.78 (0.04)	66 (1.7)
GS_Blank_20	88 (4)	4.02 (0.12)	78 (2.9)
GS_Acetate_20	87 (7)	3.93 (0.06)	85 (6)
GS_SDW_20	77 (3)	4.04 (0.18)	76 (3.3)

**Table 4:** Lag phase, maximum methane production rate, and methane yield with granular sludge and the municipal digester sludges from SDW and acetate in batch assays at 10 and 20°C.

Temperature (°C)	Substrate	Lag phase (*λ*, d)			Maximum methane production rate (*R*_m_, mL-CH_4_/d)			Methane yield (%)		
		GS	RD	VD	GS	RD	VD	GS	RD	VD
20	Acetate	0	8.9	18.1	9.7 ± 6.1	1.01 ± 0.09	0.7 ± 0.05	89 ± 6	81 ± 3	84 ± 9
	SDW	0	4	51.1	1.3 ± 0.7	0.77 ± 0.13	1.4 ± 0.4	76 ± 6	72 ± 3	67 ± 8
10	Acetate	0.75	20.1	21.7	3.5 ± 1.15	0.02 ± 0.01	0.03 ± 0.01	93 ± 2	<1	<1
	SDW	1.5	22.9	20.9	0.6 ± 0.2	0.13 ± 0.02	0.02 ± 0.01	82 ± 3	19 ± 4	<1

## Data Availability

The 16S rRNA sequences used to support the findings of this study have been deposited in the NCBI Sequence Read Archive under project SRP164945.

## Supplementary Materials

Supplementary Materials Fig. S1: soluble COD (sCOD) removal and methane yield from acetate or synthetic dairy wastewater (SDW) at (a) 20°C and (b) 10°C with different inocula (GS: granular sludge; RD: Rahola Digestate; VD: Viinikanlahti digestate). Fig. S2: principle coordinate analysis (PCoA) plot of the bacterial and archaeal classes that formed 99.9% of the microbial community in the assays inoculated with VD, RD, and GS and fed with no substrate (blank), acetate, and synthetic dairy wastewater (SDW) at 10°C and 20°C. Solid line and dashed line indicate similarity among the samples at 70 and 85%. Table S1: detailed information on the sample names used in the multivariate analysis plots.
