# Supplementary material for: Acetotrophic Activity Facilitates Methanogenesis from LCFA at Low Temperatures: Screening from Mesophilic Inocula
Source: Archaea. 2019 May 2;2019:1751783. doi: 10.1155/2019/1751783 (PMC6525847; doi:10.1155/2019/1751783)
Supplement: Supplementary Materials — Fig. S1: soluble COD (sCOD) removal and methane yield from acetate or synthetic dairy wastewater (SDW) at (a) 20°C and (b) 10°C with different inocula (GS: granular sludge; RD: Rahola Digestate; VD: Viinikanlahti digestate). Fig. S2: principle coordinate analysis (PCoA) plot of the bacterial and archaeal classes that formed 99.9% of the microbial community in the assays inoculated with VD, RD, and GS and fed with no substrate (blank), acetate, and synthetic dairy wastewater (SDW) at 10°C and 20°C. Solid line and dashed line indicate similarity among the samples at 70 and 85%. Table S1: detailed information on the sample names used in the multivariate analysis plots. [file 1751783.f1.docx]

**Acetotrophic activity facilitates methanogenesis from LCFA at low temperatures – screening from mesophilic inocula**

Suniti Singh^a^*, Johanna Rinta-Kanto^a^, Riitta Kettunen^a,b^, Piet Lens^a,c^, Gavin Collins^d^, Marika Kokko^a^ , Jukka Rintala^a^

^a^Laboratory of Chemistry and Bioengineering, Tampere University of Technology, Tampere, Finland.

^b^Present address: Tampere Water, 33800 Tampere, Finland.

^c^IHE, Institute for Water Education, Westvest 7, 2611AX Delft, The Netherlands.

^d^National University of Ireland, Galway, Ireland.

*Corresponding author: Suniti Singh Email: [suniti.singh@tut.fi](mailto:suniti.singh@tut.fi) , Tel: (+358)504478751

**Supplementary material**

(Total 1 document, 2 figures, 1 table)

Fig. S1. Soluble COD (sCOD) removal and methane yield from acetate or synthetic dairy wastewater (SDW) at (a) 20ºC and (b) 10ºC with different inocula (GS=Granular Sludge, RD=Rahola Digestate and VD=Viinikanlahti Digestate).


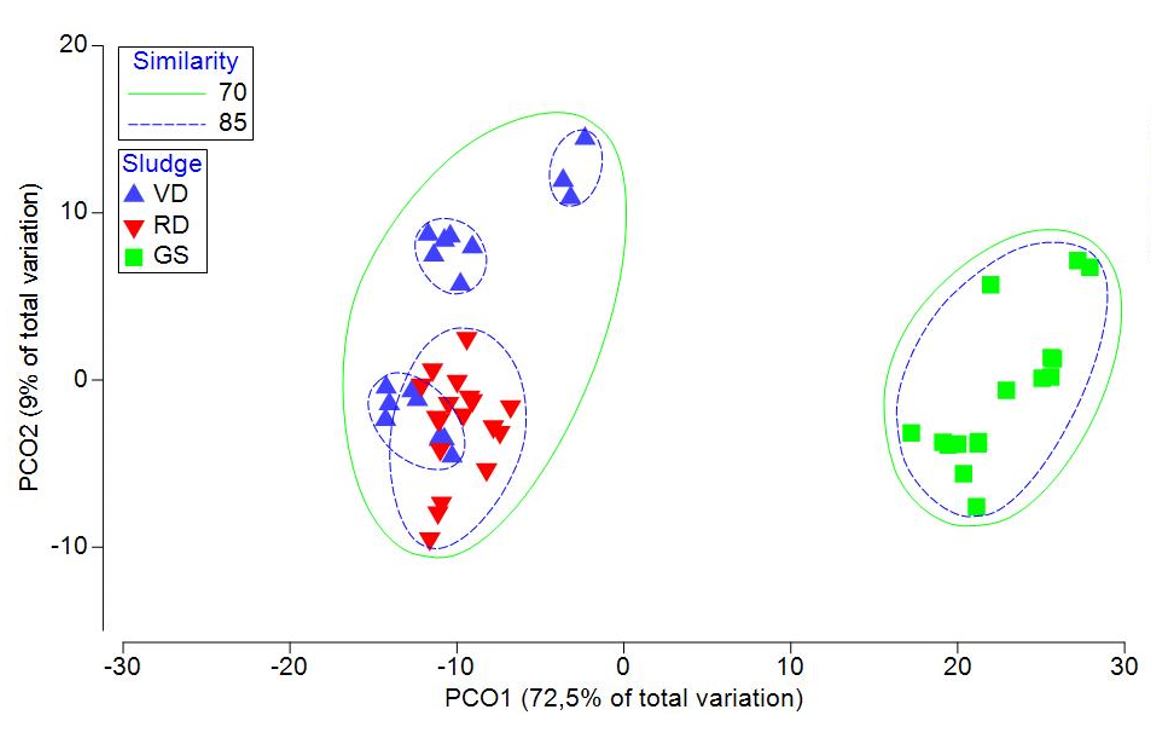


Fig. S2. Principle coordinate analysis (PCoA) plot of the bacterial and archaeal classes that formed 99.9% of the microbial community in the assays inoculated with VD, RD and GS and fed with no substrate (blank), acetate and synthetic dairy wastewater (SDW) at 10°C and 20°C. Solid line and dashed line indicate similarity among the samples at 70 and 85%.

Table S1. Detailed information on the sample names used in the multivariate analysis plots.

|  | **Inoculum** | **Temperature (°C)** | **Substrate** |
| --- | --- | --- | --- |
| VD Inoculum | VD | - | - |
| VDBlank10 | VD | 10 | Blank |
| VDAcetate10 | VD | 10 | Acetate |
| VDSDW10 | VD | 10 | SDW |
| VDBlank20 | VD | 20 | Blank |
| VDAcetate20 | VD | 20 | Acetate |
| VDSDW20 | VD | 20 | SDW |
| RD Inoculum | RD | - | - |
| RDBlank10 | RD | 10 | Blank |
| RDAcetate10 | RD | 10 | Acetate |
| RDSDW10 | RD | 10 | SDW |
| RD_Blank_20 | RD | 20 | Blank |
| RD_Acetate_20 | RD | 20 | Acetate |
| RD_SDW_20 | RD | 20 | SDW |
| GS Inoculum | GS | - | - |
| GS_Blank_10 | GS | 10 | Blank |
| GS_Acetate_10 | GS | 10 | Acetate |
| GS_SDW_10 | GS | 10 | SDW |
| GS_Blank_20 | GS | 20 | Blank |
| GS_Acetate_20 | GS | 20 | Acetate |
| GS_SDW_20 | GS | 20 | SDW |
